# Simultaneous surgery of mandibular reduction and impacted mandibular third molar extraction: A retrospective study of 65 cases

**DOI:** 10.1097/MD.0000000000019397

**Published:** 2020-04-10

**Authors:** Guodong Song, Panxi Yu, Guoqian Huang, Xianlei Zong, Le Du, Xiaonan Yang, Zuoliang Qi, Xiaolei Jin

**Affiliations:** a The 16^th^ Department, Plastic Surgery Hospital, Chinese Academy of Medical Sciences and Peking Union Medical College, Beijing; b Oral and Maxillofacial Surgery Department, Jinan Stomatological Hospital, Jinan, Shandong, China.

**Keywords:** complications, impacted mandibular third molar extraction, mandibular reduction, simultaneous surgery

## Abstract

A considerable number of patients with prominent mandibular angle have mandibular third molar impaction that needs surgical removal. Mandibular reduction is a popular and effective surgery to correct prominent mandibular angle, but it has been rarely performed simultaneously with impacted third molar extraction. In order to decrease the number of operations and suffering of patients, safely performing these 2 operations together is necessary and important. From January 2016 to June 2018, patients received mandibular reduction and impacted mandibular third molar extraction together were retrospectively reviewed. Forty-seven patients receiving long-curve mandibular reduction (n = 12) or simple mandibular reduction (n = 35) were included in this study. A total of 65 impacted mandibular third molars were extracted during mandibular reduction. One patient had hematoma within facial soft tissue which reabsorbed spontaneously. Seven patients who underwent long-curve mandibular reduction reported transient inferior lip numbness for several weeks. No infection or poor wound healing was reported. No immediate or delayed mandibular fracture occurred. All the patients were satisfied with both the aesthetic result of mandibular reduction and the unnecessity of receiving a secondary surgery to extract the impacted third molar. Simultaneously performing mandibular reduction and impacted mandibular third molar extraction can effectively reduce the number of operations and patients’ suffering. It is also safe with adequate pre-op assessment, professional surgical knowledge, proper use of surgical instruments, meticulous surgical procedures, and correct post-op care.

## 1 Introduction

As one of the most popular esthetic facial contouring surgeries in Asia, mandibular reduction is effective and highly satisfying to correct a wide and square lower face.^1^,^2^ Most patients receive this surgery in the twenties during which the mandibular development has already been completed. At this age, the impaction of mandibular third molar is also a common occurrence and its removal is the most common dentoalveolar surgical procedures.^3^,^4^

Because mandibular reduction is under general anesthesia, most patients request a simultaneous extraction of the impacted mandibular third molar. However, surgeons may suggest patients receiving these 2 operations separately to decrease operation time. Some may consider the combined therapy will increase the risk of nerve injury and mandibular fracture.^4^,^5^,^6^,^7^ According to our experience, most patients complained of not extracting the third molar during mandibular reduction at the time of follow-up, especially when general anesthesia was required in complex cases or when the impacted molar started to cause symptoms before mandibular bone healing. In order to reduce the suffering of patients, we started to perform mandibular reduction and impacted mandibular third molar extraction together, finding the 2-in-1 surgery can both safely and effectively reduce the number of operations and shorten the course of treatment. In addition, it does not increase the complication rate as compared with performing mandibular reduction alone.

## 2 Materials and methods

### 2.1 Data collection

This retrospective study was approved by the Institutional Review Board. Informed consent was obtained from the patients. From January 2016 to June 2018, 47 female patients at an average age of 24.58 ± 3.66 years were included in this study. Among them, 12 patients received long-curve mandibular reduction, and 35 patients received simple mandibular reduction. Eighteen patients received bilateral impacted mandibular third molar extraction, and 29 patients received unilateral extraction. Thus, a total of 65 impacted mandibular third molars were extracted during mandibular reduction. Other procedures including 5 advancement genioplasty, 3 maxillary subapical ostectomy, and 7 malar reduction were performed concurrently (Table 1).

**Table 1:**
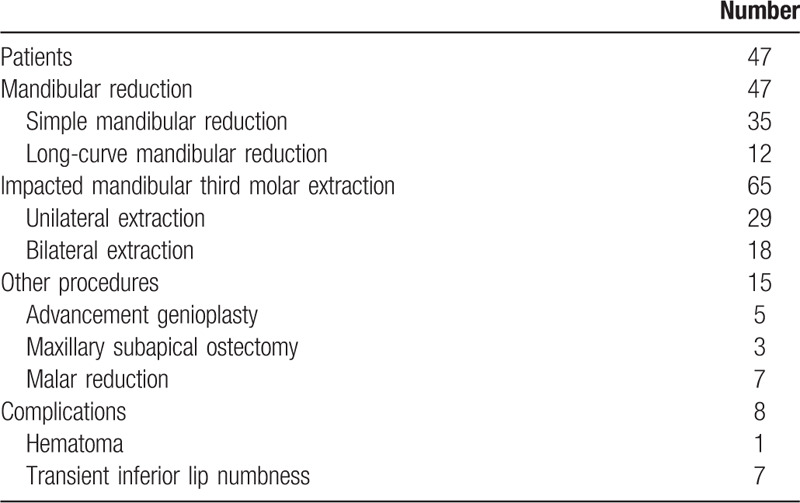
Operations performed and the related complications.

### 2.2 Pre-op evaluation

Before surgery, all the patients were evaluated by history taking, physical examination, mandibular panoramic radiographs, cephalometric x-ray, and cranial computed tomography.

The inclusion criteria were: the impacted mandibular third molar was decayed. The impacted third molar caused decay or resorption of the second molar. The impacted third molar repeatedly caused pain, pericoronitis, or food incarceration. Patients required prophylactic extraction of the impacted mandibular third molar. Patients have at least 6 months of post-op follow-up. The exclusion criteria were: patients underwent acute pericoronitis or other odontogenic infection. Patients had mandibular bone lesions such as abscesses, cysts, and tumors. Patients had any systemic disease or medication (e.g., glucocorticoids) which may impair bone strength.^[8]^ Patients had temporomandibular joint disorders, difficulty in mouth open, bleeding disorders, and any contraindication of general anesthesia. The impacted third molar occupied more than half of the mandibular height.

All the patients were informed of the underlying surgical risks and possible complications before surgery.

### 2.3 Surgical technique

**Figure 1:**
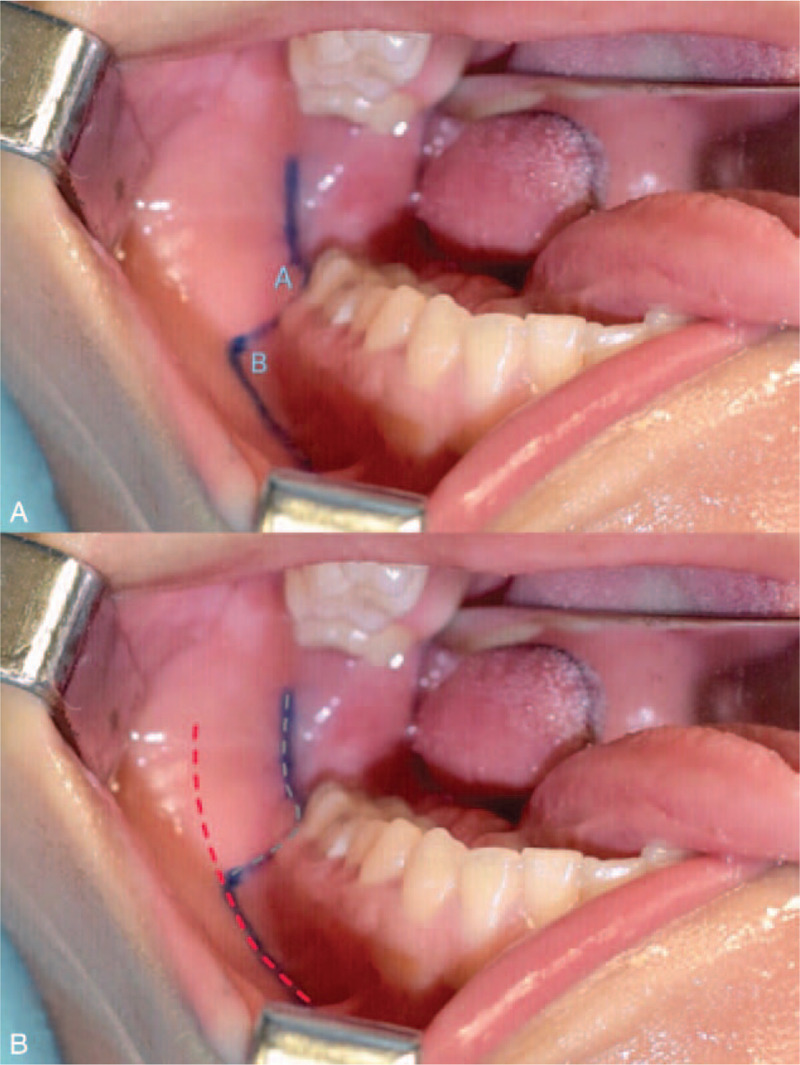
Design of the intraoral mucosal incision. The Z-shaped intraoral incision line (A) was designed as a combination of the buccally-based triangular flap incision (B, gray line) for impacted third molar extraction and the traditional incision line (B, red line) for mandibular reduction. Angle A and B should be cut into curved obtuse angles to avoid ischemic necrosis of the mucosa.

The surgery was carried out under general anesthesia by nasotrocheal intubation. In order to expose the impacted molar and the mandibular ramus, angle and body effectively, we used a Z-shaped intraoral incision derived from the buccally-based triangular flap incision for impacted third molar extraction and the traditional incision for mandibular reduction^9^,^10^ (Fig. 1). Such incision design can provide a clear surgical field and ensure sufficient blood supply for the mucosal flap. After injection of 0.5% lidocaine with 1:200,000 epinephrine along the designed incision line, mucosal incision was made with a scalpel, followed by periosteal incision with an electrotome and a subperiosteal dissection to expose the mandible.

**Figure 2:**
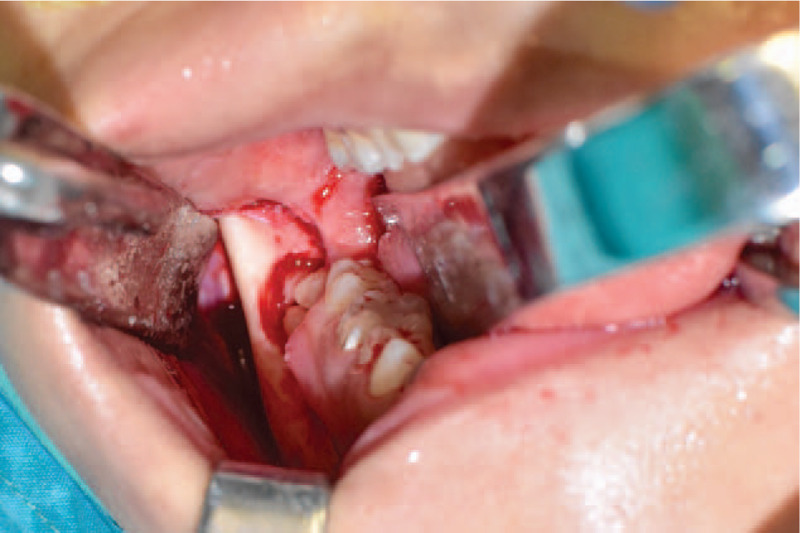
Exposure and division of the impacted mandibular third molar. A micro-drill was used to remove the surrounding bone and divide the impacted molar into several pieces for easier removal.

Mandibular bone above the impacted third molar was exposed. Some high-positioned molars could be extracted by tooth forceps directly or after being loosened by a tooth elevator without bone removal. In other conditions, bone removal was performed by a micro-drill to expose the underlying molar, which was then divided into 3 to 5 pieces by the micro-drill for easier removal (Fig. 2). The dental follicle around the crown was also removed to avoid potential pathological change. A gauze ball was used to fill the extraction wound.

After molar extraction, mandibular reduction was performed as previously described.^11^,^12^,^13^ An oval burr was used to grind the outer cortex. The grinding should be gentle around the molar extraction site to avoid accidental fracture. In simple mandibular reduction, a round burr was used to mark a curved ostectomy line from the posterior border of the ascending ramus to below the mental foramen, along which a short-neck and a long-neck oscillating saw were respectively used to saw off the mandibular outer plate and inner plate.^11^,^12^ For patients having a broad chin, a reciprocating saw was used to narrow the chin, and in this way a long-curve mandibular reduction was performed.^[13]^ The bones were completely removed after being released from attached muscles and ligaments.

After a thorough irrigation to wash out bone powders, the gauze ball filled in the extraction wound was removed. Saline was used to wash the wound gently. Fresh blood exuded from the bone was allowed to fill the extraction wound. A drainage tube along the inferior mandibular margin was kept in place for 48 hours. It should be far away from the extracted molar wound to allow natural coagulation. After wound closure, cotton dressings were applied around the lower face immediately with sufficient pressure exerted along the inferior mandibular margin.

### 2.4 Post-op management

Oral cefuroxime was routinely given for 3 days. Oral examination and cleaning were performed daily to detect infection and hematoma. Patients were discharged on the 4th post-op day after imaging examination to evaluate the immediate effect of mandibular reduction on the bone and check for fracture line and residual molar fragments. The compressive cotton dressings were replaced by an elastic mask that can provide effective protect against delayed hematoma and skin sagging. After molar extraction, delayed mandibular fracture may occur with greatest risk during post-op weeks 2 and 3, and the main cause is mastication.^[14]^ Thus, a soft diet and a masticatory-limiting force are recommended for at least 4 weeks.

## 3 Results

The follow-up duration ranged from 6 to 26 months, with a mean of 14.69 ± 5.10 months. One patient had hematoma within facial soft tissue which reabsorbed spontaneously. Seven patients who underwent long-curve mandibular reduction experienced transient inferior lip numbness for weeks. No numbness of the cheek or tongue was reported. No immediate or delayed mandibular fracture occurred. All the patients were satisfied with both the esthetic result of mandibular reduction and the unnecessity of receiving a secondary surgery to extract the impacted third molar.

### 3.1 Patient 1

**Figure 3:**
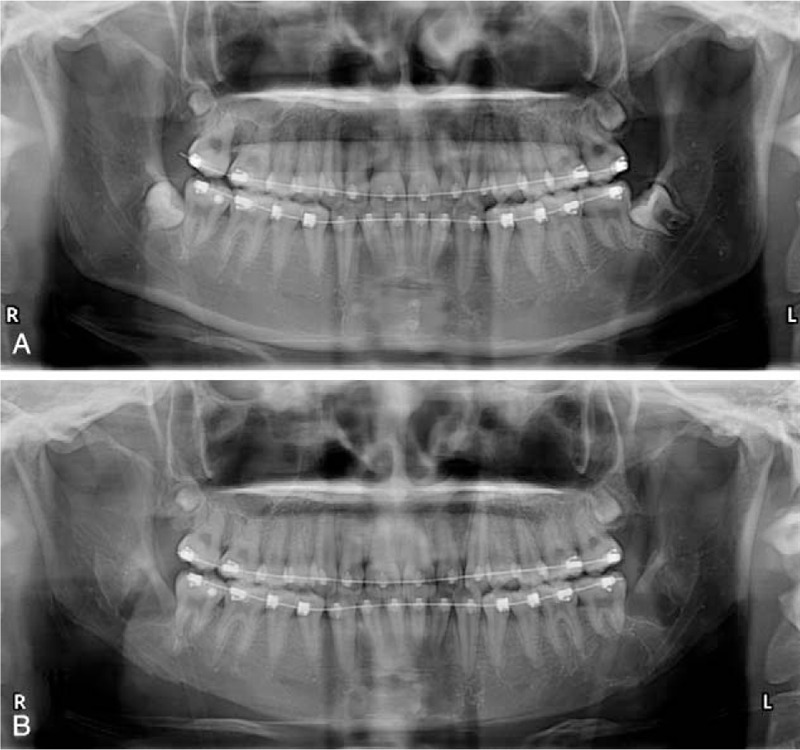
Mandibular panoramic radiographs of patient 1. The relationship among the impacted wisdom teeth, the inferior alveolar neurovascular bundle, and the inferior mandibular margin was clearly shown in the pre-op radiograph (A). Bilateral impacted wisdom teeth were extracted without fracture line in short-term post-op radiograph (B).

**Figure 4:**
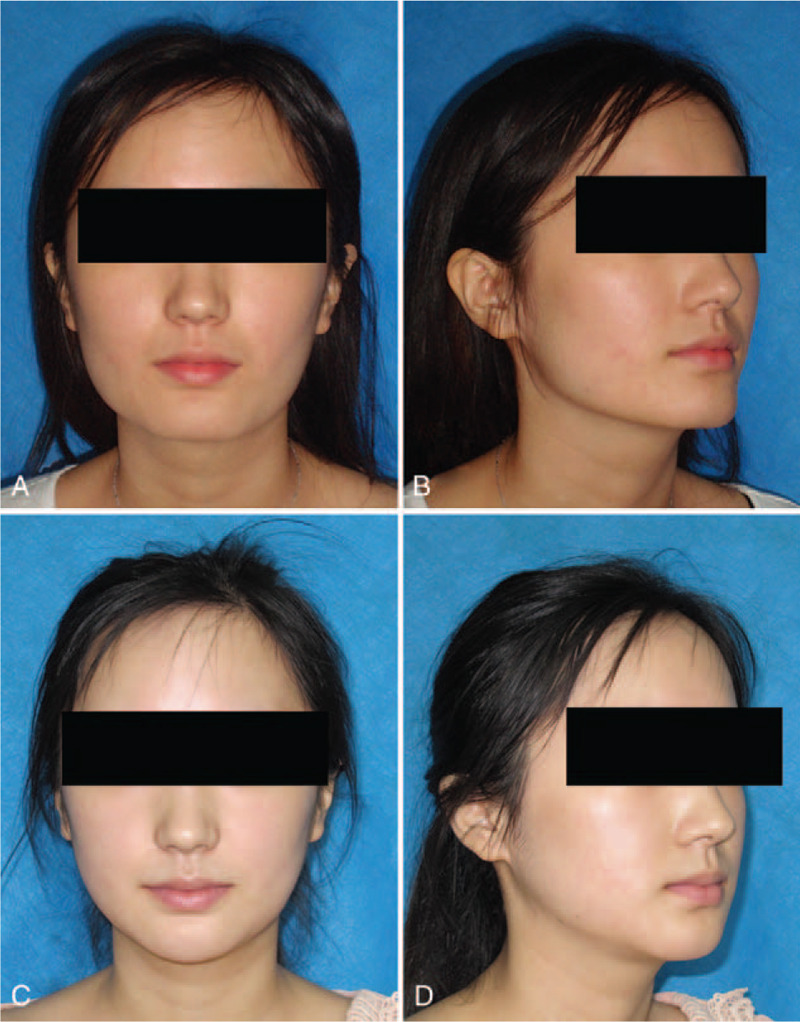
Photographs of patient 1. The patient had a square face and broad chin (A, B). After long-curve mandibular reduction, she had a new oval lower face (C, D).

A 19-year-old female patient came to our hospital to correct her square face and broad chin. The pre-op image examination showed bilateral impacted wisdom teeth that grew toward the root of the second molars (Fig. 3). Prophylactic extraction of the impacted molars was performed during long-curve mandibular reduction. The patient was satisfied with the new oval lower face and the 2-in-1 surgical procedure (Fig. 4).

### 3.2 Patient 2

**Figure 5:**
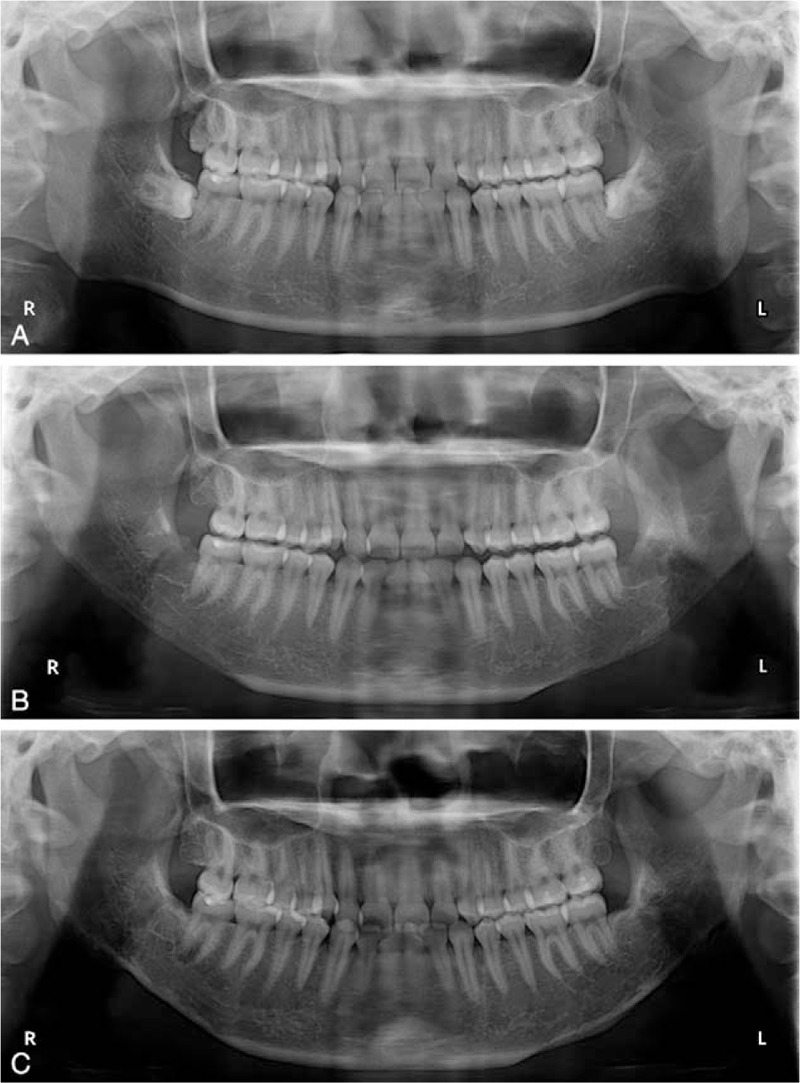
Mandibular panoramic radiographs of patient 2. The pre-op radiograph showed not only the bulged mandibular rami and body, but also the impacted wisdom teeth and right maxillary third molar (A). The short-term post-op radiograph showed that the impacted molars were completely removed without fracture line, and that the ostectomy lines were naturally curved (B). Six months after surgery, the bone healed completely (C).

**Figure 6:**
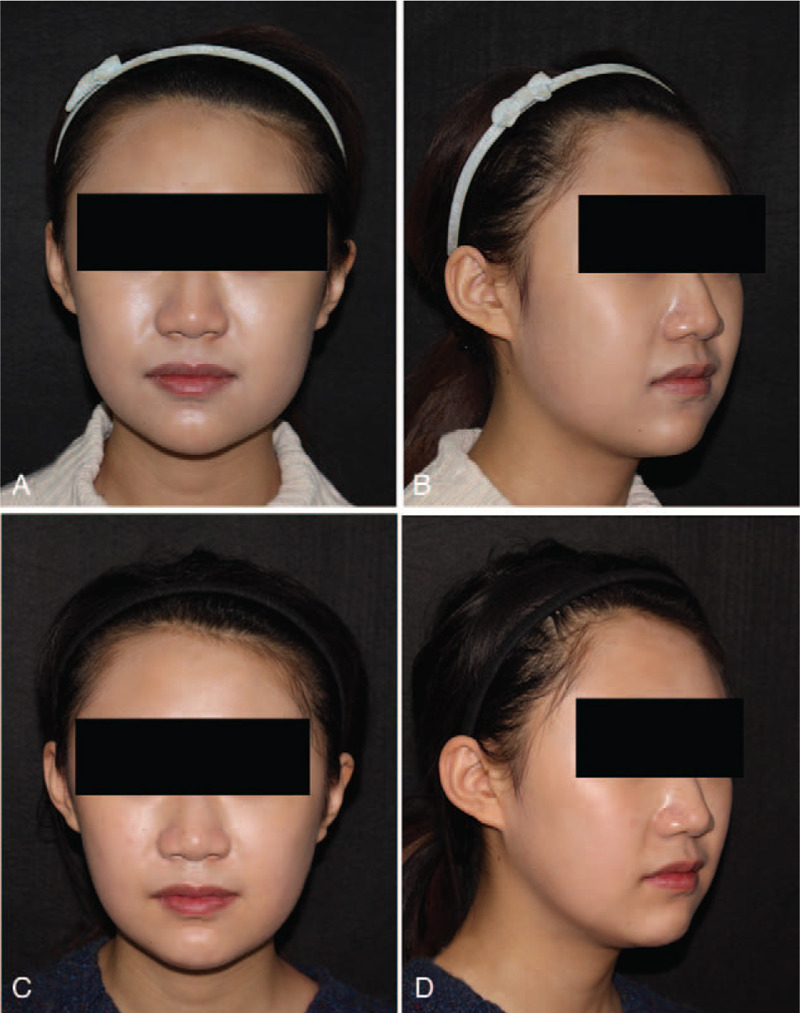
Photographs of patient 2. This patient complaining of a broad lower face received simple mandibular reduction and impacted wisdom teeth extraction (A, B). She was highly satisfied with the surgical effect (C, D).

A 25-year-old woman still had a broad lower face after type A botulinum toxin injection into the masseter. She came and asked for a slender lower face. She had no idea of the impacted teeth until the pre-op imaging examination indicated bilateral wisdom teeth impaction and right maxillary third molar impaction (Fig. 5). The patient required simultaneous molar extraction after being informed that the horizontal impacted wisdom teeth could never erupt, and their growth would cause dental crowding and damage of the second molars. Simple mandibular reduction and extraction of the impacted teeth were performed together. The patient was satisfied with the result (Fig. 6).

### 3.3 Patient 3

**Figure 7:**
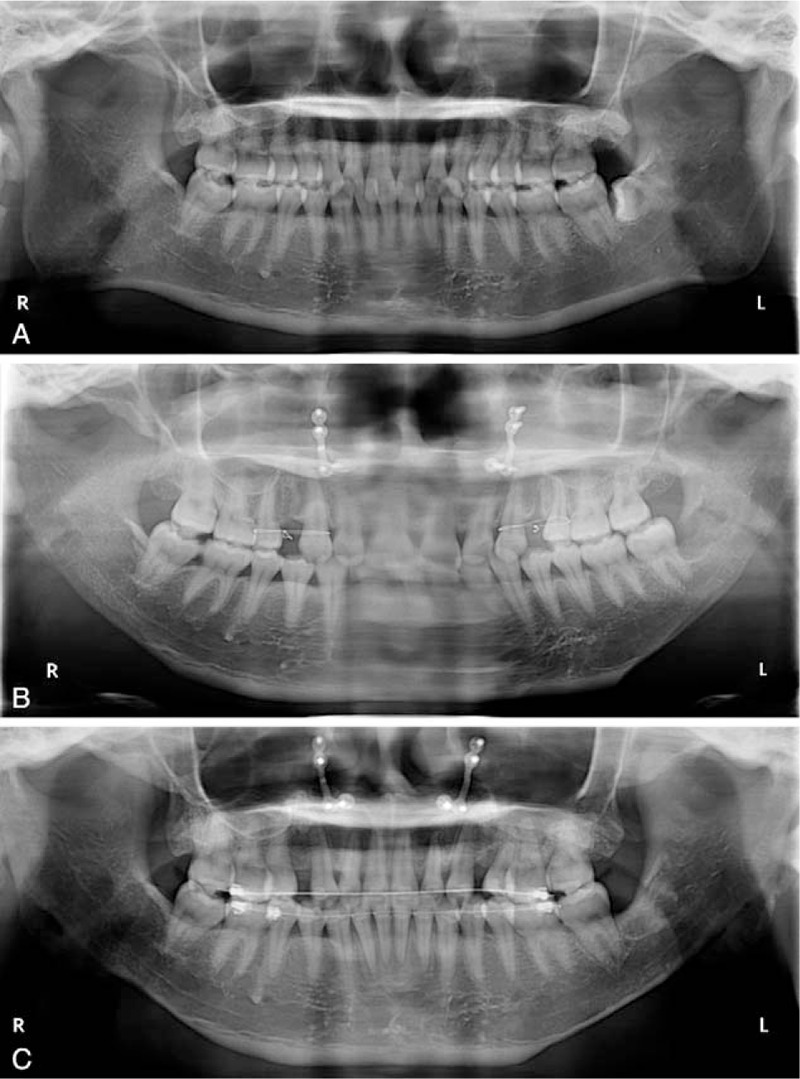
Mandibular panoramic radiographs of patient 3. Before surgery the mandibular rami were bulged with a square angle. The left wisdom tooth was horizontally impacted (A). Mandibular ostectomy was successful. The impacted wisdom tooth was extracted without fracture (B). The long-term post-op radiograph showed complete bone healing (C).

**Figure 8:**
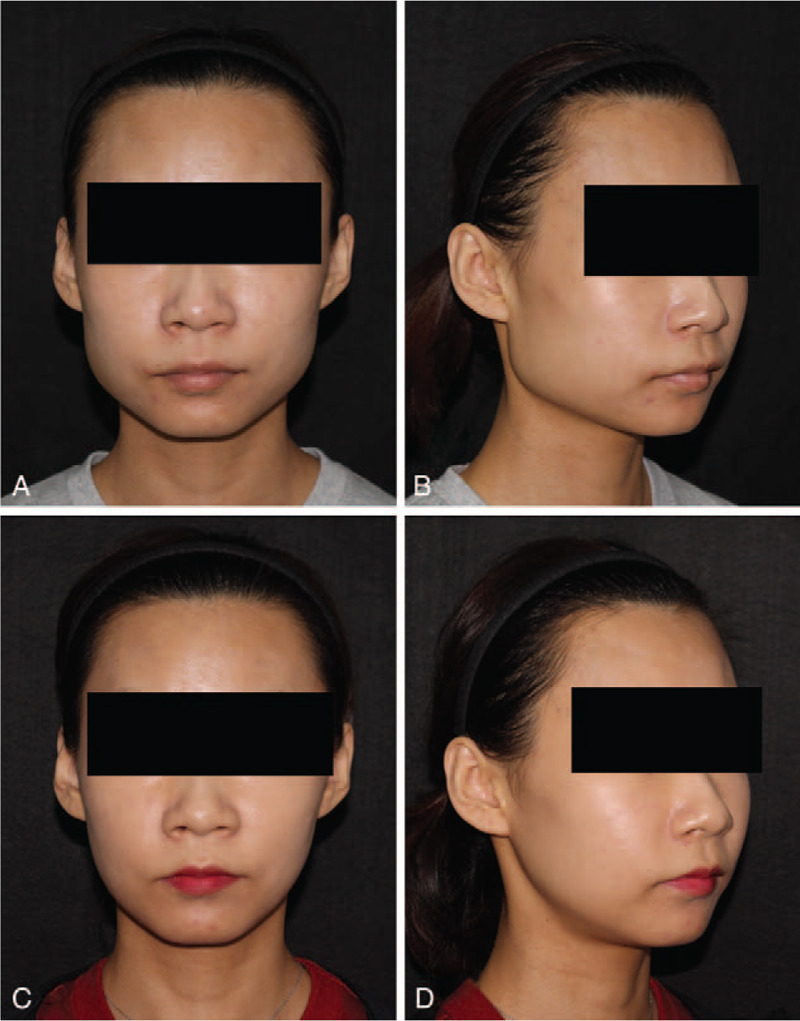
Photographs of patient 3. This patient was upset because of the asymmetrical square face (A, B). One year after surgery, the patient was very satisfied with her slender face (C, D).

The third patient was 28 years old. Her mandibular angles were protruding and asymmetrical. Her left impacted wisdom tooth was extracted before mandibular reduction (Fig. 7). More outer plate and inferior bone were removed to correct the asymmetry. Maxillary subapical ostectomy was simultaneously performed to treat the gummy smile. The 1-year post-op views show a natural oval face of this patient (Fig. 8).

### 3.4 Patient 4

**Figure 9:**
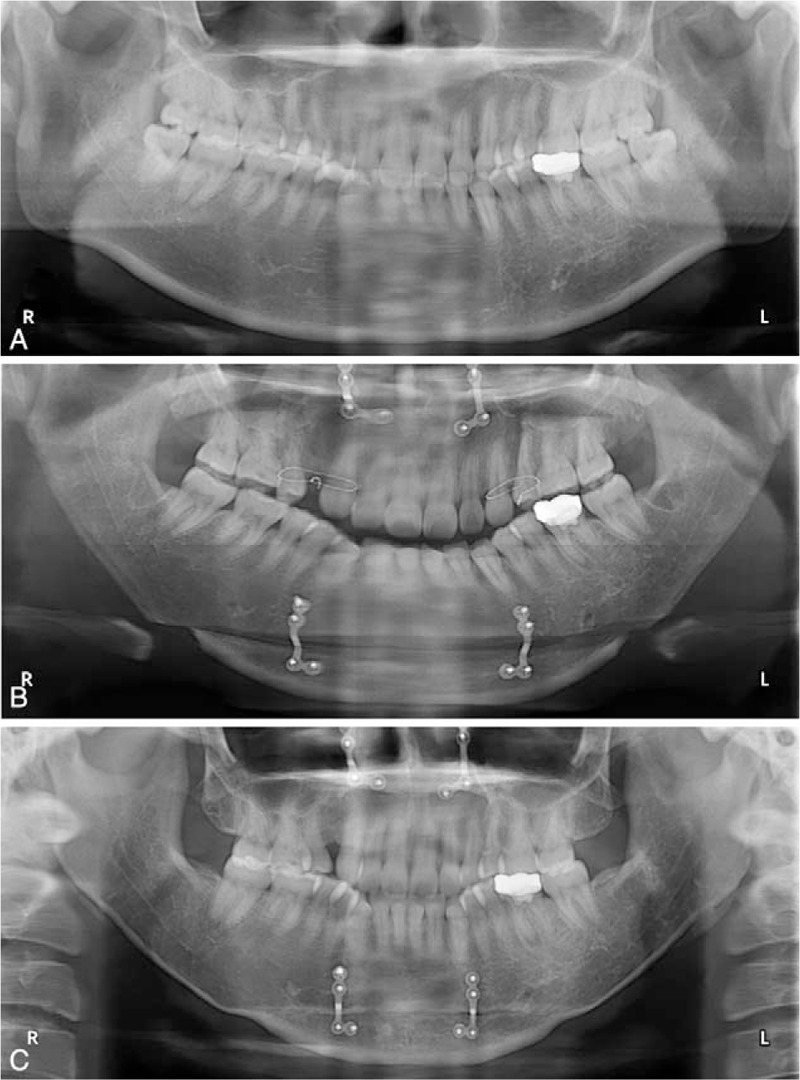
Mandibular panoramic radiographs of patient 4. Bilateral wisdom teeth were partially impacted, while the maxillary third molars had been erupted completely (A). The erupted maxillary third molars were also extracted because they would overgrow without occlusal force from the wisdom teeth (B). The 2-year post-op radiograph showed the completely healed bones (C).

**Figure 10:**
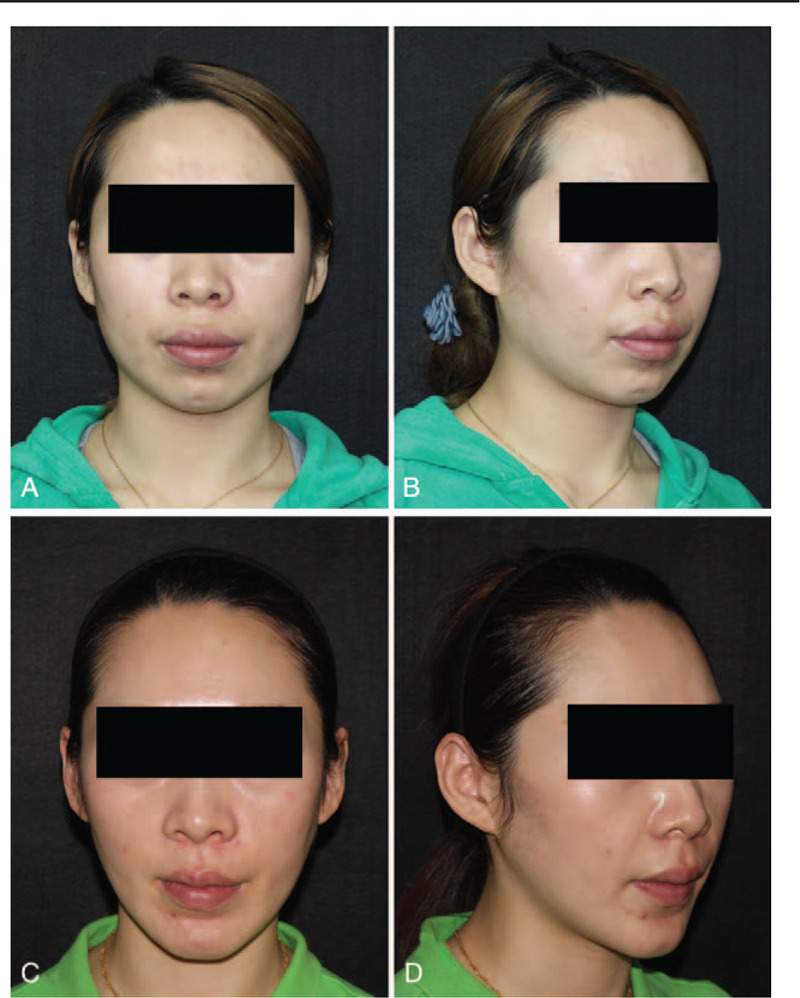
Photographs of patient 4. The patient complaining of a protruding mouth, small chin, and broad lower face had her molars extracted during facial contouring surgery (A, B). Two years after surgery, she had a completely new face contour (C, D).

A 27-year-old woman suffered from repetitive food incarceration and pericoronitis caused by the partially impacted wisdom teeth. She had hesitated for years to extract the molars because of fear. When she visited our hospital to correct her facial contour, she demanded to extract the wisdom teeth at the same time. Considering the result of physical examination and imaging examination, she was diagnosed of maxillary protrusion, prominent mandibular angle, microgenia, and bilateral wisdom teeth impaction (Figs. 9 and 10). Maxillary subapical ostectomy, molar extraction, simple mandibular reduction, and advancement genioplasty were successively performed. The patient recovered uneventfully and was grateful to have a new face contour.

## 4 Discussion

Although both mandibular reduction and impacted wisdom teeth extraction have risks of wound infection, mandibular fracture, and inferior alveolar neurovascular bundle injury, performing them together does not mean doubling the complications. In our center, the therapeutic strategies from patient selection, to surgical procedure, complication prevention, and post-op care have been improved to ensure the safety of this 2-in-1 surgery.

Identifying patients that present a higher risk of complications is the key to avoid complications. Before surgery, patients were strictly selected according to the aforementioned inclusion and exclusion criteria. The relationship among the impacted third molar, the inferior alveolar neurovascular bundle, and the inferior mandibular margin is vitally important and can be clearly evaluated by the pre-op mandibular panoramic radiograph (Figs. 3, 5, 7 and 9). Osseous occupation is the hypothesized mechanism by which mandibular third molar increases the risk of mandibular fracture.^[15]^ The deeper the level of impaction is, the greater the surgical difficulty is.^[16]^ The fracture risk is awfully increased when third molar occupies more than half of the mandibular height,^[17]^ which is therefore one of the exclusion criteria of patient selection. The lower-located impacted molar also makes it difficult to remove enough bone mass to achieve a satisfactory contour reshaping effect, although in patients with prominent mandibular angle, the inferior mandibular margin is generally at a distance from the inferior alveolar neurovascular bundle and impacted molar.

Mandibular fracture perhaps is the most severe implication that can occur in either mandibular third molar extraction^[7]^ or mandibular reduction.^[5]^ However, third molar extraction is associated with angle fracture while mandibular reduction is associated with subcondylar fracture. Therefore, the notion that the combination of these 2 procedures doubles the risk of mandibular fracture is not persuasive. So far, there is only 1 case reporting iatrogenic fracture during mandibular reduction and wisdom teeth extraction.^[18]^ In this study, the authors extracted the third molar after mandibular reduction, and the fracture occurred during the attempt to loosen the molar using a dental elevator. There were 2 possible reasons for this iatrogenic fracture: the removal of mandibular bone, including the outer cortex and the angle, had seriously weakened the bony strength around the impacted molar. The use of a dental elevator to repeatedly loosen the molar exerted considerable force in the direction of the mandibular angle, making this area more prone to fracture. In our practice, we extracted the impacted molar first. During molar extraction, excessive force should not be placed on the bone, especially in the direction of mandibular angle. The elevator was only used for high-positioned molars that did not require or required little bone removal. The osteotome was never used for bone chiseling or molar division, because it exerted repeated sudden forces on the mandibular angle. Instead, a high-speed micro-drill was applied to conservatively remove a necessary amount of bone for molar exposure and divide the molar for easier removal (Fig. 2).

Curved ostectomy usually does not cause fracture of mandibular angle if the distance between the ostectomy line and extraction site was sufficiently evaluated before surgery. Instead, the occurrence rate of subcondylar fracture is higher during ostectomy. And it usually occurs when the oscillating saw proceeds upward rather than backward because of the limited exposure of the posterior border of the mandibular ramus. In our practice, there are 3 means to reduce the risk of mandibular subcondylar fractures. First, fully expose the posterior margin of the ascending ramus; second, ostectomy should not exceed the level of occlusal plane; third, use a long-neck oscillating saw with a long tip to cut the posterior border by leaning on the saw, so that an effective cutting in the desired direction can be achieved.

Neurosensory deficit occurs in 1% to 8% of patients who undergo extraction of an impacted mandibular third molar.^[4]^ The inferior alveolar nerve, buccal nerve, and lingual nerve may be irritated or damaged, mainly manifesting as the numbness of the ipsilateral lower lip, cheek, and tongue. The control of complications mainly relies on pre-op imaging evaluation to determine the positional relationship between the molar and the nerves, and to avoid exerting forces in the direction of the nerves. It is more necessary to gently extract the molar and avoid scratching the alveolar bone if the molar is close to the inferior alveolar nerve. Transient lower lip numbness due to irritation of the mental nerve is common after mandibular ostectomy. The incidence varies from 4.7% to 44.4% according to the range of mandibular ostectomy.^10^,^13^ Direct mental nerve rupture is rare as the nerve can be clearly seen by the surgeon. In this study, all the 7 patients who experienced transient inferior lip numbness underwent long-curve mandibular reduction, which itself alone has a very high risk of mental nerve irritation as the saw just passes under the nerve during ostectomy.

A major limitation of this study comes from the variation among surgeons in the judgment on the need for prophylactic removal of impacted mandibular third molars. Although asymptomatic disease-free impacted third molars may be associated with increased risk of periodontitis and potential pathological changes in the long term, insufficient evidence is available to determine whether or not they should be removed.^[19]^ Given the lack of available evidence, the decision to extract impacted third molars or not should be individualized rather than generalized, and patient values should be considered.^19^,^20^ In our study, the pre-op evaluation, molar extraction, and mandibular reduction were performed by the same maxillofacial surgeon who has a solid foundation in oral surgery, to guarantee the necessity and safety of the simultaneous molar extraction. And all the patients agreed with the operative plan.

## 5 Conclusions

Performing mandibular reduction and impacted mandibular third molar extraction together can effectively reduce the number of operations and anesthesia, eliminating patients’ fear of the secondary surgery. This 2-in-1 surgery is also safe with full pre-op assessment, adequate surgical expertise, proper use of surgical instruments, meticulous surgical procedures, and correct post-op care.

## Acknowledgment

The authors are very grateful to the secretary of their department, Ms. Xiaoyu Hou, for her help in clinical data storage and everything she does in daily work. Otherwise, all the authors declare that they have no financial or personal interference with other people or organizations that could influence their work.

## Author contributions

Guodong Song and Panxi Yu made substantial contributions to conception and design, collected the clinical data and wrote the manuscript. Guoqian Huang, Xianlei Zong, Le Du, Xiaonan Yang participated the surgeries performed in this study. Zuoliang Qi revised the manuscript. Xiaolei Jin performed the surgeries, generally supervised this study, and gave final approval of the version to be published. All authors read and approved the final manuscript.
